# Investigation of the efficacy and safety of lung biopsy plus microwave ablation for a solitary suspected malignant pulmonary nodule after radical mastectomy

**DOI:** 10.3389/fonc.2025.1525114

**Published:** 2025-08-13

**Authors:** Chao Xing, Peishun Li, Sen Yang, Qirong Man, Xusheng Zhang, Qianqian Yuan, Miaomiao Hu, Yunling Bai, Kaixian Zhang

**Affiliations:** Department of Oncology, Tengzhou Central People’s Hospital, Tengzhou, Shandong, China

**Keywords:** lung biopsy, microwave ablation (MWA), pulmonary nodule, breast cancer, lung metastases

## Abstract

**Purpose:**

To evaluate the safety and efficacy of CT-guided lung biopsy combined with microwave ablation (MWA) for solitary suspected malignant pulmonary nodules in post-radical surgery breast cancer patients.

**Materials and methods:**

This retrospective study included 37 post-radical surgery breast cancer patients with solitary suspected malignant pulmonary nodules, treated with CT-guided lung biopsy and MWA between January 2014 and December 2018. Institutional review board approval was obtained. Clinical outcomes and complications were analyzed.

**Results:**

Pathological results identified primary lung cancer in 5 patients (13.5%, 5/37) and metastatic invasive ductal carcinoma (breast origin) in 30 patients (81.1%, 30/37). Major complications included pneumothorax (n=8, 21.6%), chest pain (n=6, 16.2%), and hemoptysis (n=4, 10.8%). For metastatic cases, 2-, 3-, and 5-year survival rates were 86.2%, 58.3%, and 35.3%, respectively. The median progression-free survival after MWA was 35 months (range: 4–72; 95% CI: 24.53–46.48), and median overall survival was 44 months (95% CI: 32.55–55.45).

**Conclusion:**

CT-guided lung biopsy combined with MWA is a safe and effective approach for managing solitary suspected malignant pulmonary nodules in post-radical surgery breast cancer patients.

## Introduction

Breast cancer is one of the most common prevalent tumors among women, with a mortality-to-incidence ratio of 15% (1). Lung metastases are frequently witnessed in breast cancer patients (2). As a result, when intrapulmonary nodules are detected in breast cancer patients, they are frequently misdiagnosed as lung metastases. Nevertheless, studies have indicated that the incidence of primary lung cancer in breast cancer patients is approximately 1% (3–5), while the incidence of concurrent double primary cancer (with a time difference between diagnoses of no more than 6 months) is about 0.6% in breast cancer patients (4). Identifying a solitary pulmonary nodule in patients with breast cancer poses a diagnostic challenge. For such nodules, surgical resection is a feasible option. However, many patients are either unable or unwilling to undergo surgery due to factors such as advanced age, poor cardiopulmonary function, or other reasons. Recent studies (6–10) have demonstrated that lung biopsy combined with microwave ablation (MWA) for the solitary pulmonary nodule can yield outcomes similar to surgical resection.

However, there is scarce research exploring the application of this technology in breast cancer patients who have undergone radical surgery and subsequently developed a solitary pulmonary nodule. To fill this gap, we conducted a retrospective study to assess the efficacy of a concurrent diagnostic and therapeutic approach. This approach entailed conducting a CT-guided biopsy, immediately followed by MWA of the solitary pulmonary nodule suspected of malignancy in patients with a history of radical breast cancer surgery.

## Materials and methods

### Subjects

This retrospective study included 37 patients who underwent CT-guided lung biopsy combined with microwave ablation for suspected malignant solitary pulmonary nodules after radical mastectomy from January 2014 to December 2018. All patients had histopathologically confirmed invasive ductal carcinoma and underwent radical mastectomy. Chest computed tomography (CT) imaging demonstrated the existence of a newly identified solitary pulmonary nodule. The baseline imaging comprised chest and abdominal computed tomography (CT), enhanced cranial MRI, whole-body bone scan ECT, and, if accessible, positron-emission tomography (PET) CT. All patients were regarded as ineligible for reoperation or declined to undergo surgical resection.

Exclusion criteria encompassed the following: (1) Uncontrolled infectious inflammation around the lesion; (2) Skin infection or ulceration at the puncture site; (3) Severe pulmonary fibrosis, especially drug-induced fibrosis (11, 12); (4) Patients with a marked bleeding propensity and coagulation disorders; (5) Cachexia; (6) Severe cardiopulmonary insufficiency.

**Figure 1:**
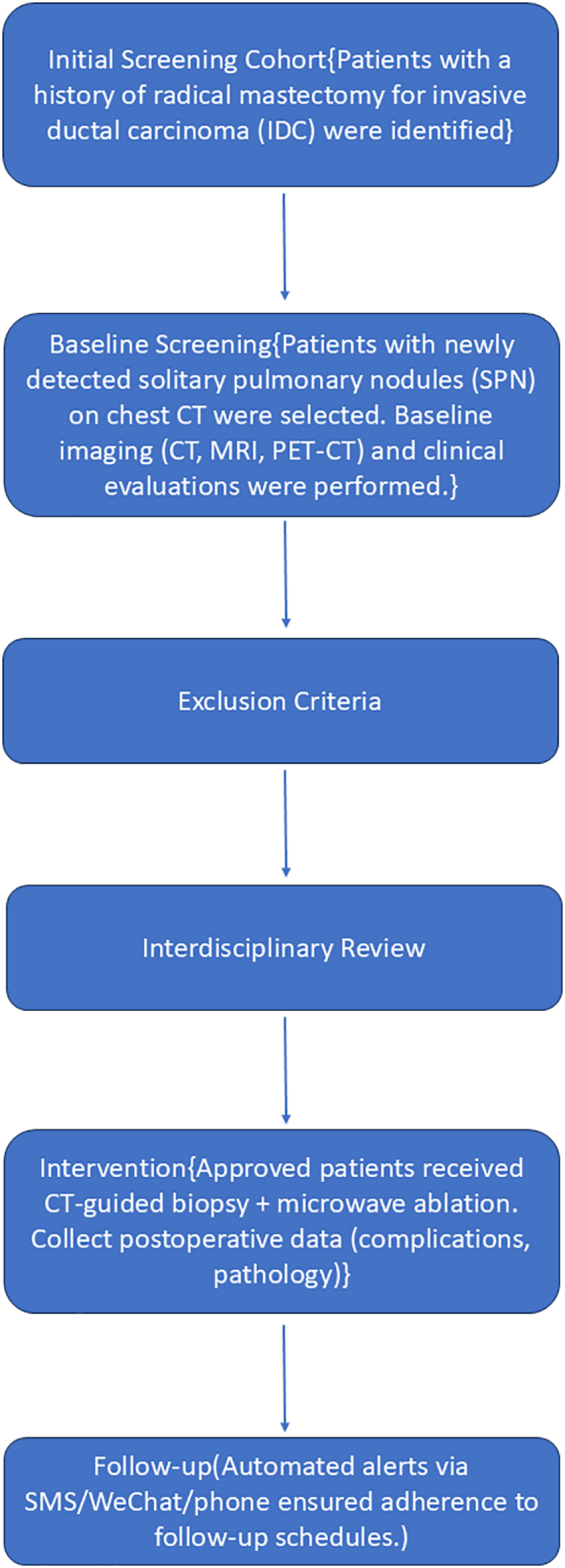
Flow chart of the eligibility process for the study.

All cases were reviewed by an interdisciplinary oncology committee consisting of thoracic surgeons, respiratory physicians, medical oncologists, radiation oncologists, diagnostic and interventional radiologists, pathologists, and anesthesiologists. The flow chart is shown in **Figure 1**.

### Instrumentation

A Siemens Light Speed 64V spiral CT scanner (Germany) guided biopsy and MWA. Under CT guidance, transthoracic core biopsies used an 18G Argon coaxial system (MCXS1820LX semi-automatic). MWA employed an ECO-100A1 system (SFDA 20172011470; Nanjing ECO) with 2,450 ± 20 MHz frequency and 0-150W adjustable power. The 16G-20G microwave antenna (150-200mm length) featured a 15-mm active tip and water-cooled system to reduce surface temperature.

### Procedure of the operation

Prior to treatment, patients underwent thorough clinical evaluation, including laboratory tests, imaging, and pulmonary function assessments. Blood work included coagulation studies. Anticoagulants were held 1 week pre-procedure to minimize bleeding risk.

Patients were positioned supine or prone based on nodule location, secured with a vacuum-negative pressure pad, and the puncture site was marked on the skin.

An 18-gauge biopsy core needle was inserted into the center of the tumor through a coaxial cannula before initiating MWA. A biopsy was performed first to obtain two or three specimens from a single core needle. The tissue samples were preserved in 10% formalin and later evaluated pathologically after H&E staining. All biopsy specimens underwent immunohistochemical testing (including ER, PR, and HER-2). A CT scan was done to monitor for biopsy-related complications.

Under CT guidance, the MWA probe was accurately positioned in the pulmonary tumor. Limited pneumothorax without progression during MWA was acceptable. However, chest tube insertion was required for progressive pneumothorax interfering with probe placement or causing clinical symptoms. Ablation power was typically 30-50W for 3-10 minutes. CT scans monitored probe targeting, adjusted depth/angle, and ensured the intended ablation zone. Continuous monitoring of vital signs (BP, HR, ECG, SpO_2_) was performed throughout the procedure.

An immediate post-MWA CT scan frequently displayed ground-glass opacity (GGO) 0.5 to 1.0 cm in width at the periphery of the pulmonary nodule, indicating complete ablation (13, 14). The CT scan was also employed to assess for complications such as pneumothorax, hemothorax, or pleural effusion. If a progressive pneumothorax was detected, chest tube insertion would be indicated to manage the situation.

This retrospective study was approved by the Ethics Committee of Tengzhou Central People’s Hospital (Ethics Review No. 2018-Ethics Review-08). All participants provided written informed consent after detailed explanation of the procedures.

### Assessment of therapeutic efficacy and follow-up

Patients underwent enhanced CT scans at 1, 3, 6, and 12 months after MWA in the first year, and then every 6 months thereafter. Enhancement at the lesion site was considered indicative of incomplete treatment. Regions that remained unenhanced and were larger than the treated metastases were regarded as representing complete ablative necrosis and thus considered fully effective for the treatment.

The primary response rate was defined as the percentage of target tumors successfully eliminated during the initial ablation session. The assessment of the local efficacy of MWA was conducted by a single oncologist and two radiologists.

Survival outcomes included progression-free survival (the time from MWA until the recurrence of other metastases or death, PFS) and overall survival (the time from MWA until death, OS).

### Statistical analysis

IBM SPSS 26.0 was used for statistical analysis. Data are presented as the total count (percentage) and mean values. The Chi-square test was utilized for categorical variables. The Kaplan-Meier method was used to determine the survival rate and local progression-free survival rate. In all statistical assessments, results were regarded as significant if p < 0.05.

## Results

### General information

From January 2014 to December 2018, 37 female patients with a solitary suspected malignant pulmonary nodule after radical breast cancer surgery were treated with CT-guided lung biopsy combined with MWA in our hospital. All patients were female and had undergone modified radical mastectomy with pathologically confirmed invasive ductal carcinoma. The median age was 53 years (range: 27 to 73 years). HER-2 was detected by immunohistochemistry in all patients, and 10 were strongly positive (verified by FISH). 34 patients received adjuvant chemotherapy, 11 received adjuvant radiotherapy, 10 received adjuvant targeted therapy, and 22 received adjuvant endocrine therapy. The pulmonary nodules ranged in size from 6 to 28 mm (15.65 ± 6.13) (**Table 1**).

**Table 1:** Characteristics of patients.

Characteristics	n (%)
Total number of patients	37
Age (years)	
<60	27 (73.0%)
≥60	10 (27.0%)
Previous chemotherapy	
No	3 (8.1%)
Yes	34 (91.9%)
Previous radiotherapy	
No	26 (70.3%)
Yes	11 (29.7%)
Previous endocrine therapy	
No	15 (40.5%)
Yes	22 (59.5%)
ER	
Negative	15 (40.5%)
Positive	22 (59.5%)
PR	
Negative	16 (43.2%)
Positive	21 (56.8%)
HER-2 over-expression	
No	27 (73.0%)
Yes	10 (27.0%)
Maximum tumor diameter (cm)	
≤ 1.0	10 (27.0%)
1.0<~≤2.0	17 (46.0%)
2.0<~≤3.0	10 (27.0%)

ER, estrogen receptor; PR, progesterone receptor; HER-2: human epidermal growth factor receptor2.

All patients underwent technically successful lung biopsy combined with MWA. One month after the operation, a CT scan showed that 37 lesions were completely covered by the tumor coagulation area after ablation, and the primary effective rate was 100% (37/37).

### Pathological results of puncture biopsy

**Figure 2:**
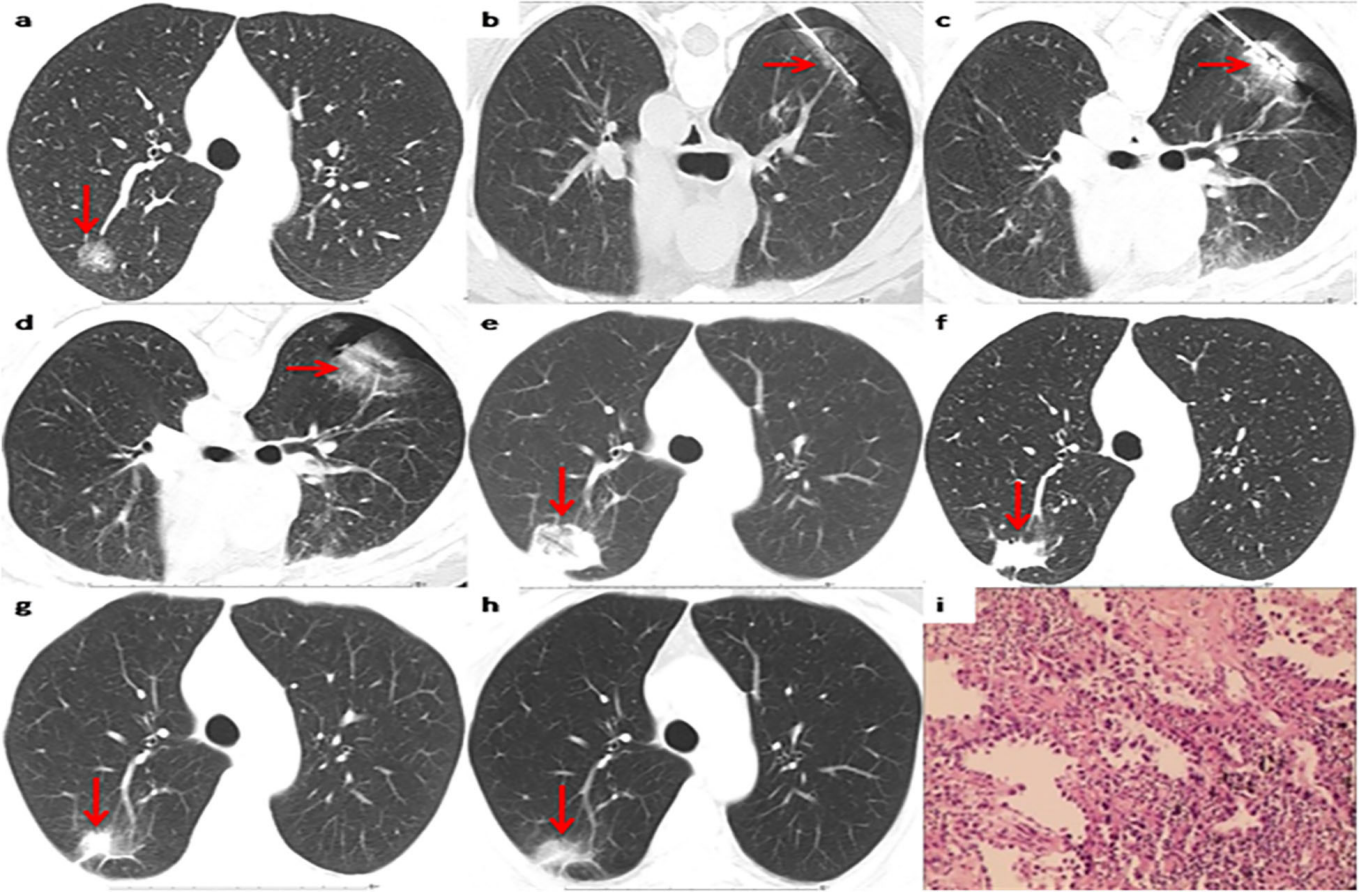
Representative CT scans of a patient with primary lung invasive adenocarcinoma after breast cancer surgery. **(a)** Preoperative image. **(b)** The biopsy needle has been inserted into the nodule center to complete the biopsy sectioning. **(c)** The MWA antenna has been inserted into the nodule center. **(d)** Postoperative image immediately after MWA. **(e)** 1 month after MWA. **(f)** 12months after MWA. **(g)** 36 months after MWA. **(h)** 60 months after MWA. **(i)** Pathological results of primary lung invasive adenocarcinoma biopsy.

**Figure 3:**
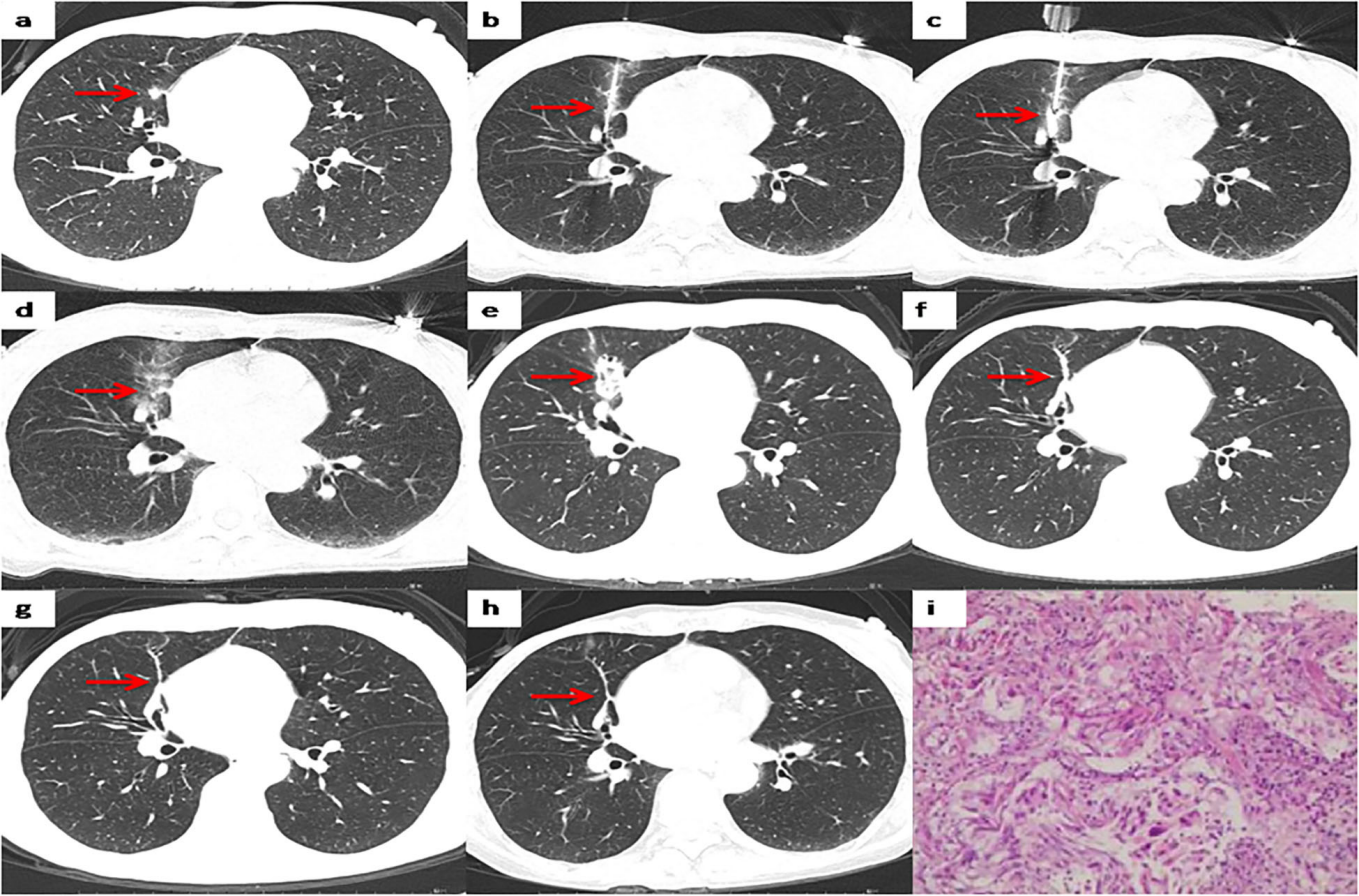
Representative CT scans of a patient with right lung metastases after breast cancer surgery. **(a)** Preoperative image. **(b)** The biopsy needle has been inserted into the nodule center to complete the biopsy sectioning. **(c)** The MWA antenna has been inserted into the nodule center. **(d)** Postoperative image immediately after MWA. **(e)** 1 month after MWA. **(f)** 12months after MWA. **(g)** 36 months after MWA. **(h)** 60 months after MWA. **(i)** Pathological results of lung metastases biopsy.

Among the 37 patients, 35 cases (35/37, 94.6%) were pathologically diagnosed as malignant tumors, among which 5 cases (5/37, 13.5%) were diagnosed as primary lung cancer (**Figure 2**). The biopsy pathology of 30 cases (30/37, 81.1%) was invasive ductal carcinoma (**Table 2**, **Figure 3**). The remaining 2 cases (2/37, 5.4%) were diagnosed as atypical adenomatous hyperplasia (AAH) (**Table 2**). A separate subgroup analysis was conducted for the clinical treatment of the 30 cases with lung metastases from breast cancer.

**Table 2:** Pathological results of puncture biopsy.

Histopathology results	Number	%
Total	37	
Invasive Ductal Carcinoma	30	81.1%
Atypical Adenomatous Hyperplasia	2	5.4%
Adenocarcinoma In Situ	1	2.7%
Invasive Adenocarcinoma	3	8.1%
Minimally Invasive Adenocarcinoma	1	2.7%

### Postoperative complications

The major complications were pneumothorax, chest pain, and hemoptysis. Pneumothorax occurred in 11 of 37 cases (29.7%). Severe (lung compression >50%) and moderate (lung compression 20%-50%) pneumothorax occurred in two cases, and these patients underwent catheter drainage. The other nine patients’ pneumothorax was gradually absorbed without special treatment. The incidence of chest pain was 73.0% (27/37), and that of hemoptysis was 27.0% (10/37). Among the 10 hemoptysis cases, three were moderate (hemoptysis volume 10-100 mL), seven were mild (hemoptysis volume <10 mL), and there was no severe hemoptysis (hemoptysis volume >100 mL). No other complications such as needle implantation metastases, pulmonary embolism, or bronchopleural fistula were observed (**Table 3**).

**Table 3:** Side effects and complications during and after microwave ablation procedure*.

Side effects and complications	n (%)
Pneumothorax	
Grade 1	9 (24.3%)
Grade 3	2 (5.4%)
Chest pain	
Grade 1	23 (62.6%)
Grade 2	4 (10.8%)
Hemoptysis	
Grade 1	7 (18.9%)
Grade 2	3 (8.1%)
Fever	
Grade 1 (38°C-39°C)	2 (5.4%)
Fatigue	
Grade 1	4 (10.8%)
Pleural effusion	
Grade 1	8 (21.6%)
Nausea	
Grade 1	9 (24.3%)
Vomiting	
Grade 1	3 (8.1%)
Cough	
Grade 1	11 (29.7%)

*Complications were graded according to the Common Terminology Criteria for Adverse Events (CTCAE) version 5.0.

### Postoperative PFS of lung metastases subgroup

During follow-up, local progression at the ablation site (Local Tumor Progression, LTP) occurred in 16.7% (5/30) of cases during a median follow-up of 44 months. These five lesions were from five different patients, two of whom underwent a second ablation treatment and the other three who opted for medical therapy. The 1-year, 2-year, and 3-year cumulative LTP rates were 3.3%, 10.0%, and 16.7%, respectively.

The median time from MWA of lung metastases to disease progression was 35 months (ranged 4-72 months, 95% confidence interval 24.53–46.48). Univariate analysis indicated that the PFS after MWA was related to time from primary tumor to lung metastases, HER-2 over-expression, and histological grade (P<0.05) (**Table 4**). Cox regression analysis demonstrated that time from the primary tumor to lung metastases and histological grade had a significant effect on PFS (P<0.05).

**Table 4:** PFS after MWA according to lung metastatic tumor and treatment.

Prognostic factors	N	Median PFS (months)	95% CI	χ 2	p value
The size of lung metastatic tumor (cm)				2.195	0.334
0 ≤ 1	8	35	24.84-45.16		
1.0<~≤2.0	14	30	14.32-45.68		
2.0<~≤3.0	8	17			
Initial TNM staging				0.194	0.908
I	3	34	0-70.81		
II	15	39	20.08-57.92		
III	12	35	21.60-48.40		
ER				0.589	0.443
Positive	16	35	26.84-43.16		
Negative	14	30	24.00-36.00		
Time from primary tumor to lung metastases (months)				5.445	0.020
<60	18	27	15.63-38.37		
≥60	12	45	40.22-49.78		
HER-2 over-expression				5.849	0.016
Yes	9	45	33.05-56.95		
No	21	27	10.85-43.15		
Histological grade				10.267	0.006
I	8	45	39.15-50.85		
II	12	34	24.10-43.91		
III	10	15	10.00-20.00		

CI, confidence interval.

### Postoperative OS of lung metastases subgroup

The 2-year, 3-year and 5-year survival rates were 86.2%, 58.3% and 35.3% respectively. The median OS time in the lung metastases subgroup was 44 months (95% confidence interval 32.55–55.45). Univariate analysis revealed that OS was related to the time from primary tumor to lung metastases, HER-2 over-expression and histological grade (P<0.05) (**Table 5**). Cox regression analysis demonstrated that HER-2 over-expression and histological grade had a significant effect on OS (P<0.05) (**Table 6**).

**Table 5:** OS after MWA according to lung metastatic tumor and treatment.

Prognostic factors	N	Median OS (months)	95% CI	χ 2	p value
The size of lung metastatic tumor (cm)				3.034	0.219
0 ≤ 1	8	63	35.28-90.72		
1.0<~≤2.0	14	39	26.87-51.13		
2.0<~≤3.0	8	28	25.91-30.09		
Initial TNM staging				0.955	0.620
I	3	64	0.00-148.82		
II	15	39	24.54-53.46		
III	12	44	17.40-70.61		
ER				0.932	0.334
Positive	16	44	35.58-52.42		
Negative	14	35	12.65-57.35		
Time from primary tumor to lung metastases (months)				5.932	0.015
<60	18	35	22.86-47.14		
≥60	12	63	49.68-76.32		
HER-2 over-expression				9.009	0.003
Yes	9	65	42.19-87.81		
No	21	35	21.01-48.99		
Histological grade				11.173	0.004
I	8	63	24.45-101.55		
II	12	39	27.31-50.69		
III	10	25	2.49-47.51		

**Table 6:** Multivariate analysis of PFS.

Prognostic factors	PFS			OS		
	Odds ratio	95% CI	p	Odds ratio	95% CI	p
Time from primary tumor to lung metastases (months)	3.864	1.014-14.726	0.048	3.590	0.941-13.694	0.061
HER-2 over-expression	2.586	0.791-8.454	0.116	7.090	1.399-35.936	0.018
Histological grade	2.418	1.044-5.600	0.039	3.142	1.239-7.971	0.016

## Discussion

In the context of a history of breast cancer, a solitary pulmonary nodule could potentially be lung metastases, primary lung cancer, or benign lung lesions (15). According to a review (16), the incidence of metastatic lesions ranged from 34% to 75%, that of primary lung cancer varied from 12% to 48%, and for benign lesions, it was from 14% to 18%. This study, focusing on CT-guided lung biopsy combined with MWA for suspected malignant nodules, found primary lung cancer in 13.5% (5/37) and breast cancer metastases in 81.1% (30/37), yielding a 94.6% malignancy rate (35/37)—higher than historical data. This phenomenon may be related to the following reasons: (1) The breast cancer TNM stage of the patient group included in this study at the initial treatment was relatively late; (2) tissue sampling via biopsy improving diagnostic accuracy; (3) Small sample size with selection bias; (4) The included patients had a longer follow-up time and regular periodic examinations, enabling earlier detection of malignant lesions and increasing the detection rate of malignant tumors.

In 2021, WHO histological classification of lung tumors defined atypical adenomatous hyperplasia and adenocarcinoma *in situ* (AIS) as glandular prodromal lesions (17). Asymptomatic slow-growing glandular prodromal lesions can be managed conservatively with careful observations and regular follow-up. Even after surgical treatment, the 5-year disease-free survival rate of patients after complete surgical resection of AIS is 100% or close to 100% (18). In this study, 3 cases of glandular prodromal lesions were not only pathologically diagnosed but also inactivated by thermal ablation after synchronous diagnosis and treatment. While conservative observation is typical for such lesions, the protocol’s synchronous biopsy-ablation approach prioritized timely intervention, aligning with the patients’ high-risk profile and the procedure’s demonstrated safety.

For patients with advanced lung metastases of breast cancer, systemic treatment such as chemotherapy, hormonal therapy, and anti-HER2 are main methods of therapy. There is currently no consensus on whether solitary lung metastases need surgery. Friedel et al. (19) reported 467 patients with lung metastases from breast cancer, of which 84% patients underwent complete resection, and the 5 -, 10 -, and 15-year survival rates were 38%, 22%, and 20%, respectively. According to the International Lung Metastases Registry (20), the median OS and 5-year OS rates in patients undergoing surgical resection of lung metastases from breast cancer were 37 months and 38% in the R0 group. As a minimally invasive technique, local thermal ablation has been applied to the treatment of early lung cancer, and the number of lung cancer patients treated each year is rapidly increasing (21–24). It has been proved that percutaneous thermal ablation can also effectively treat lung metastases (25–28). In this study, the 5-year survival rate of patients with lung metastases who underwent needle biopsy combined with MWA was 35.3%, which was similar to that reported in previous studies. These findings may be explained by three key factors. First, all breast cancer patients with lung metastases received personalized multimodal therapy post-MWA, integrating chemotherapy, endocrine therapy, targeted agents, and immunotherapy as indicated. Second, MWA effectively debulked local tumors, reducing the risk of systemic spread. Third, comprehensive pre-treatment staging excluded extrapulmonary disease in all enrolled patients.

In this study, the median time from MWA of lung metastases to disease progression was 35 months (range 4–72 months, 95% CI 24.53–46.48). Univariate analysis revealed that post-MWA PFS correlated significantly with time from primary tumor to lung metastases, HER-2 overexpression, and histological grade (P<0.05, **Table 4**). Cox regression showed time from primary to lung metastases and histological grade significantly affected PFS (P<0.05, **Table 6**), confirming them as independent prognostic factors for local control in breast cancer patients with lung metastases. The median overall survival (OS) time in the lung metastases subgroup was 44 months (95% confidence interval 32.55–55.45). Univariate analysis showed that OS was related to the time from primary tumor to lung metastases, HER-2 over-expression, and histological grade (P<0.05, **Table 5**). Cox regression showed HER-2 overexpression and histological grade significantly impacted OS (P<0.05, **Table 6**), confirming them as independent prognostic factors for breast cancer patients with lung metastases. This study identified time from primary to lung metastases, histological grade, and HER-2 status as key prognostic factors in breast cancer lung metastases. These findings warrant validation in larger studies.

Needle biopsy and MWA have similar procedures and complications (pneumothorax, bleeding, etc.) (29–31). Complications are closely related to the physiological conditions of lung tissue and the times of pleural puncture (32). Chi et al. (33) reported that the incidence of pneumothorax was 25%in coaxial biopsy combined with MWA for ground-glass nodes. In this study, the incidence of pneumothorax was 29.7%, which was higher than previously reported in the literature. Wang et al. (34) reported that the incidence of hemoptysis following pulmonary nodule ablation was 22%. In this study, the incidence of hemoptysis was 27%, which was higher than previously reported in the literature. These differences may be because most patients in this study had pulmonary nodules with a maximum diameter less than 2cm (73.0%). Due to small nodule size, multiple adjustments of the biopsy needle are needed for accuracy, and all patients require 2-3 biopsies. These multiple operations increase the risk of damage to lung tissues and blood vessels, raising the incidence of hemoptysis.

This study has inherent limitations. As a retrospective analysis, it relies on pre-existing records, susceptible to incomplete data, inaccuracies, and selection bias. The small sample size compromises statistical power, limiting generalizability. Notably, no comparative analysis was performed with alternative modalities (surgical resection, SBRT, RFA). The lack of head-to-head comparisons hinders definition of MWA’s clinical role. Prospective, multicenter randomized controlled trials with larger cohorts are needed to rigorously assess MWA’s efficacy and safety profile.

In conclusion, this study shows that concurrent lung biopsy with MWA demonstrates significant clinical value for suspected malignant pulmonary nodules, enabling simultaneous diagnosis and therapeutic intervention. However, patient selection and optimal treatment timing remain key challenges in clinical implementation.

## Data Availability

The original contributions presented in the study are included in the article/supplementary material. Further inquiries can be directed to the corresponding author.
